# Evidence for endothelial‐to‐mesenchymal transition in human brain arteriovenous malformations

**DOI:** 10.1002/ctm2.99

**Published:** 2020-06-21

**Authors:** Lorelei D. Shoemaker, Aaron K. McCormick, Breanna M. Allen, Steven D. Chang

**Affiliations:** ^1^ Stanford Neuromolecular Innovation Program Department of Neurosurgery Stanford University Stanford California; ^2^ Department of Microbiology & Immunology University of California San Francisco California

**Keywords:** arteriovenous malformation (AVM), cerebrovascular disease, endothelial‐to‐mesenchymal transition (EndMT), myofibroblast

## Abstract

**Background:**

Brain arteriovenous malformations (AVMs) are rare, potentially devastating cerebrovascular lesions that can occur in both children and adults. AVMs are largely sporadic and the basic disease biology remains unclear, limiting advances in both detection and treatment. This study aimed to investigate human brain AVMs for endothelial‐to‐mesenchymal transition (EndMT), a process recently implicated in cerebral cavernous malformations (CCMs).

**Methods:**

We used 29 paraffin‐embedded and 13 fresh/frozen human brain AVM samples to profile expression of panels of EndMT‐associated proteins and RNAs. CCMs, a cerebrovascular disease also characterized by abnormal vasculature, were used as a primary comparison, given that EndMT specifically contributes to CCM disease biology. AVM‐derived cell lines were isolated from three fresh, surgical AVM samples and characterized by protein expression.

**Results:**

We observed high collagen deposition, high PAI‐1 expression, and expression of EndMT‐associated transcription factors such as KLF4, SNAI1, and SNAI2 and mesenchymal‐associated markers such as VIM, ACTA2, and S100A4. SMAD‐dependent TGF‐β signaling was not strongly activated in AVMs and this pathway may be only partially involved in mediating EndMT. Using serum‐free culture conditions, we isolated myofibroblast‐like cell populations from AVMs that expressed a unique range of proteins associated with mature cell types and with EndMT. Conditioned medium from these cells led to increased proliferation of HUVECs and SMCs.

**Conclusions:**

Collectively, our results suggest a role for EndMT in AVM disease. This may lead to new avenues for disease models to further our understanding of disease mechanisms, and to the development of improved diagnostics and therapeutics.

**Abbreviations**
AVM: arteriovenous malformation
AVM‐CM: arteriovenous malformation cell line conditioned medium
CCM: cerebral cavernous malformation
EC: endothelial cells
ECM: extracellular matrix
EndMT: endothelial‐to‐mesenchymal transition
HHT: hereditary hemorrhagic telangiectasia
HUVEC: human umbilical vein endothelial cells
HUVEC‐CM: human umbilical vein endothelial cell conditioned medium
NB: normal brain
SMC: smooth muscle cells
SMC‐CM: smooth muscle cell conditioned medium

## 1 INTRODUCTION

Brain arteriovenous malformations (AVMs) are cerebrovascular lesions that occur throughout the brain, vary in size and blood flow, and can lead to seizures, headaches, progressive neurological deficits, and hemorrhage in both children and adults.^1^, ^2^ AVMs are characterized by a lack of a capillary bed resulting in direct arterial‐venous communication, and by the ambiguous arterial/venous/lymphatic identity of their endothelial cells (ECs).^3^ Although promising work has identified somatic mutations in KRAS in AVM tissue, there is no known genetic cause, limiting the availability of research tools.^4^, ^5^

AVMs are dynamic, are known to regrow following treatment, and are capable of proliferating for at least 120 days following xenograft transplantation into nude mice.^6^ We and others have previously reported proliferating Nestin‐ and Ki67‐positive vascular and perivascular cells and expression of lymphatic‐ and proliferation‐associated proteins such as COUP‐TFII, PROX1, and LYVE1.^3^, ^7^, ^8^, ^9^, ^10^, ^11^ Although there is evidence for an altered microenvironment within the AVM, the precise identities of all cell types present in AVMs, and their interactions, remain elusive.^12^ The mechanisms of disease are also unclear and although no single signaling pathway currently defines AVMs, activation of MAPK‐ERK signaling and angiogenic pathways such as Notch, Thrombospondin, and VEGF have been implicated.^3^, ^5^, ^13^, ^14^, ^15^, ^16^

Endothelial‐to‐mesenchymal transition (EndMT) has been recently found to contribute to inherited and sporadic cerebral cavernous malformations (CCMs) in humans, a disease characterized by dilated and irregular cerebrovasculature, similar to but distinct from AVMs.^17^, ^18^ EndMT is a process whereby mature ECs acquire the characteristics of mesenchymal cells, marked by proliferation and invasiveness, disorganization of EC junctions, and a spindle‐like morphology. There is a loss of expression of EC markers, such as PECAM, and increased expression of mesenchymal markers such as α‐smooth muscle actin (α‐SMA), fibroblast‐specific protein‐1 (FSP‐1 or S100A4), Vimentin, and extracellular matrix (ECM) proteins .^19^ The TGF‐β pathway plays a major role in regulating EndMT in cardiovascular, pulmonary, and hepatic development and disease, and has been implicated in CCM‐associated EndMT.^19^ Mutations in genes involved in TGF‐β signaling also result in hereditary hemorrhagic telangiectasia (HHT), a vascular disease with a high incidence of brain AVMs but likely distinct from sporadic AVMs.^20^, ^21^

Our study focused on determining the presence of EndMT, using various techniques, in 43 unique human brain AVMs. We found evidence through the expression of EndMT‐associated transcription factors (TFs) and mesenchymal markers including KLF4, SNAI1/2, VIM, ACTA2, and S100A4. We observed high collagen deposition and expression of PAI‐1, an inhibitor of fibrinolysis that is primarily transcriptionally regulated by TGF‐β. However, we observed limited expression of SMAD4 and phosphorylated SMADs (pSMADs), suggesting that SMAD‐dependent TGF‐β signaling may not be the primary driver of EndMT and PAI‐1 expression in this cellular context. Using serum‐free culture conditions, we isolated myofibroblast‐like cell populations from AVMs that expressed a range of proteins including ACTA2, PAI‐1, and S100A4. Conditioned medium from these cells resulted in dramatic phenotypic changes in HUVECs and increased proliferation of normal HUVECs and SMCs in vitro.

## 2 MATERIALS AND METHODS

### 2.1 Human AVM, CCM, and control brain tissue

Patient demographics and clinical histories are summarized in Table S1. Samples were obtained during surgery by the Department of Neurosurgery, Stanford. All patients provided written, informed consent and the study was performed in compliance with HIPAA and the regulations outlined by the Stanford University Institutional Review Board Administrative Panels for the Protection of Human Subjects (Protocol #49290). There was no clinical history of venous malformation‐associated syndromes, such as HHT. AVM and CCM samples were paraffin‐embedded by the Neuropathology Department or were fresh frozen (FF) and stored at –80°C for later analysis. A total of 43 unique AVM samples were examined in the following manner: five archived FF samples for qRT‐PCR (labeled AVM‐1‐5), eight archived FF samples for western blot (labeled AVM‐33‐40), 29 archived formalin‐fixed paraffin‐embedded (FFPE) samples for immunohistochemistry (IHC) (labeled AVM‐6‐32, AVM‐1, and AVM‐5), and three surgically fresh samples for tissue culture (labeled AVM‐A‐C). Eight unique sporadic CCM samples were examined in the following manner: four archived FF samples for qRT‐PCR (labeled CCM‐1‐4) and four archived FFPE samples for IHC (labeled CCM‐5‐8). One FFPE and one FF normal human brain cortical samples were obtained from the Stanford Cancer Center Tissue Bank. To validate antibodies and qRT‐PCR primers, various FFPE and postmortem‐frozen human tissues obtained from the Tissue Bank were used, including human epileptic cortex, heart, liver, spleen, lung, adrenal gland, kidney, and lymph nodes.

### 2.2 qRT‐PCR

As previously described,^3^ RNA was extracted with an RNeasy Plus Mini kit and a QIAshredder column (Qiagen). cDNA was synthesized using BioRad iScript RT Supermix, and qRT‐PCR reaction mixtures were prepared with TaqMan Gene Expression Master Mix (Applied Biosystems). A standard program was used with the BioRad CFX96 Real‐Time qPCR System. Primer efficiency was determined for all primers (Applied Biosystems), a list of which is in Table S2. Assays were performed in triplicate for each unique sample, with *CT* variation ≤0.5, using GAPDH as a reference gene. Values are reported as the mean of 2^Δ^ *^CT^* ± standard deviation (SD). Samples with *CT* values >37 were considered not detected (ND).

### 2.3 Immunohistochemistry

IHC was performed as previously described.^3^ Briefly, 4‐μm sections of paraffin‐embedded samples were de‐paraffinized, and exposed to appropriate heat‐mediated antigen retrieval. Sections were exposed to primary antibodies at 4°C O/N, and secondary antibodies at RT for 1 h. DAB (3,3′‐diaminobenzidine, Vector Labs) was used to visualize immunoreactivity and Mayer's Hematoxylin (MilliporeSigma) was used to indicate nuclei. The following antibodies were used: PECAM (monoclonal rabbit, 1:300, Millipore), PAI‐1 (polyclonal rabbit, 1:100, Sigma Prestige), SNAI1/2 (polyclonal rabbit, 1:200, Abcam), S100A4 (polyclonal rabbit, 1:200, Sigma Prestige), SMAD4 (monoclonal rabbit, 1:400, Cell Signaling Technology), and secondary antibodies (antirabbit, 1:200, Vector Labs). The Trichrome assay (Abcam) was used according to manufacturer's specifications to evaluate collagen deposition. Slides were imaged using a Zeiss Axio Imager M2 microscope.

### 2.4 Western blot

FF samples from eight AVMs were lysed on ice in RIPA buffer containing 1 mM DTT and protease/phosphatase inhibitor cocktail (MilliporeSigma), using a Qiagen TissueRuptor. Homogenates were centrifuged at 4°C for 5 min and the supernatant was removed and stored at –20°C. Protein concentration was determined using the BCA assay (Pierce/ThermoScientific), and protein loading was confirmed using Vinculin expression (monoclonal rabbit, 1:5000, Abcam EPR8185). A total of 50 μg of protein lysate per sample was loaded and run on BioRad TGX MiniPROTEAN 4‐15% gels, transferred to PVDF membrane using the BioRad Semi‐dry Trans‐Blot system. Following blocking in 5% BSA in TBST, the membranes were probed with primary antibodies at 4°C O/N and secondary antibodies for 1 h at RT. The antibodies used were the following: SMAD4 (mAb38454), SMAD3 (mAb9523), pSMAD3 (mAb9520), SMAD2 (mAb5339), pSMAD2 (mAb3108), SMAD5 (mAb12534), SMAD1 (mAb6944), pSMAD1/5 (mAb9516), and antirabbit secondary (Ab7074) (Cell Signaling). Performance and specificity of the p/SMAD antibodies were tested on commercially available p/SMAD2/3‐positive controls as well as on mouse TGF‐β‐activated brain lysate. Proteins were detected using ECL Chemiluminescence (GE Healthcare) and the membranes were imaged using a BioRad Universal Hood III Imager, with no postimage processing.

### 2.5 Generation of human brain AVM cell lines

The AVM cell lines were isolated using an adapted protocol for the serum‐free isolation of rodent neural progenitors.^22^ Human AVM tissue was placed in Neurobasal‐A (NBA) media (Gibco) within 12 h of surgery, rinsed with DMEM (Gibco), and chopped into small pieces with heavily cauterized tissue removed. The tissue was enzymatically dissociated with papain (MilliporeSigma), dispase (Roche), and DNase (Worthington) enzyme mixture for 30 min at 37°C, with mixing and trituration every 15 min. Following centrifugation, the cell pellet was resuspended in defined NBA, subjected to a Percoll gradient (GE Healthcare), centrifuged for 15 min at 1500 rpm and the cell pellet was rinsed three times in defined NBA. Cells were cultured in serum‐free NBA supplemented with 2% B27 (Gibco), 1% Glutamax (Gibco), and 40 ng/mL each of EGF and FGF (Peprotech) at 37°C in a humidified chamber (5% CO_2_) at 10^4^ cells/mL on fibronectin (CalbioChem)/PDL (MilliporeSigma)‐coated plates. After 48 h, the medium was changed, with the nonattached cells centrifuged and replated. For long‐term propagation, 75% of the medium was changed every 2‐3 days and the cells were passaged at 80% confluence. Bright field images were captured on the Zeiss Axio Observer A1. NBA AVM‐conditioned medium (NBA AVM‐CM) used for other experiments was prepared from medium that was exposed to AVM cell lines for 48 h, centrifuged, pooled, and stored at –20°C.

### 2.6 Human cell lines

Commercially available HUVECs and aSMCs were cultured according to manufacturer's guidelines (Lonza) and not used beyond P9. Attached neural progenitors (SD56) were used for comparison only.

### 2.7 Immunocytochemistry

Cell lines were grown at 1 × 10^4^ cells/cm^2^ on PDL‐coated glass coverslips for 48 h, fixed with 4% paraformaldehyde, and exposed to primary antibodies O/N at 4°C and to secondary antibodies for 2 h at RT. The following antibodies were used: PECAM (polyclonal rabbit, 1:20, Abcam), PAI‐1 (polyclonal rabbit, 1:50, Sigma Prestige), SNAI1/2 (polyclonal rabbit, 1:500, Abcam), S100A4 (monoclonal mouse, 1:80, Sigma), αSMA (monoclonal rabbit, 1:500, Abcam; monoclonal mouse, 1:100, Sigma), and AlexaFlour secondary antibodies (1:1500, ThermoFisher Scientific). Hoechst 33342 was used to label cell nuclei. Slides were imaged using a Zeiss Axio Imager M2 microscope.

### 2.8 Proliferation

HUVECs and aSMCs (P7‐9) were cultured in EGM‐2 and SmGM medium, respectively, for 24 h. The cells were rinsed twice in prewarmed PBS, and three separate conditions were tested: normal growth medium (EGM‐2 for HUVECs and SmGM for aSMCs), NBA growth medium, and NBA growth medium exposed to AVM cell lines (NBA AVM‐CM). Cells were cultured for 24 h and 5‐ethynyl‐2′‐deoxyuridine (EdU) was added 4 h prior to the end of the experiment for HUVECs and 6 h prior for aSMCs. Cell proliferation determined the Click‐iT Edu incorporation assay according to manufacturer's instructions and the nuclei were identified with Hoechst 33342 dye (Invitrogen/Molecular Probes). The assays were performed in triplicate, with four randomly chosen images obtained from each using a Zeiss Axio Imager M2 microscope. Total cells (Hoechst‐positive) and total proliferating cells (Hoechst‐ and Edu‐positive) were counted for all images.

### 2.9 Conditioned medium luminex: eBioscience/Affymetrix Magnetic 63‐plex Cytokine Array

SMCs (P5) and HUVECs (P6) were grown in SmGM and EGM‐2, respectively. Cells were rinsed with PBS followed by NBA, and cultured in NBA growth medium for 48 h. The media was collected, centrifuged, and aliquoted and stored at –20°C. Aliquots of NBA HUVEC‐CM, NBA SMC‐CM, NBA AVM‐CM, and NBA growth medium were analyzed using the Luminex‐ eBioscience/Affymetrix Magnetic 63‐plex Cytokine Array, in the Stanford Human Immune Monitoring Center using adapted manufacturer's recommendations. Beads were added to a 96‐well plate and washed in a Biotek ELx405 washer. Samples were added to the plate containing the mixed antibody‐linked beads, incubated at RT for 1 h and then at 4°C O/N with shaking. The plate was washed, exposed to biotinylated antibody for 75 min at RT, and then exposed to streptavidin‐PE for 30 min at RT, followed by reading buffer. Each sample was measured in duplicate. Plates were read using a Luminex 200 instrument with a lower bound of 50 beads per sample per cytokine. Custom assay Control beads by Radix Biosolutions were added to all wells.

### 2.10 Statistics

*T*‐tests were used to determine statistical significance unless otherwise noted. The significance level was set at *P* < .05.

## 3 RESULTS

### 3.1 AVMs express EndMT‐associated molecules

**FIGURE 1:**
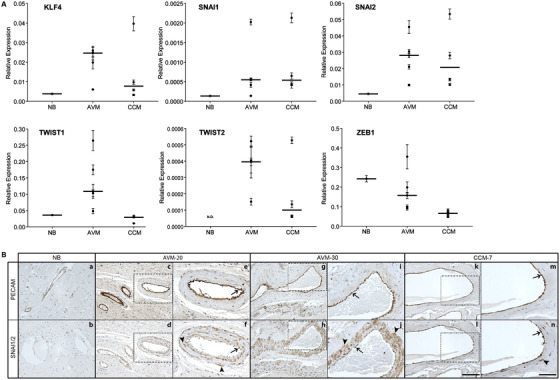
EndMT‐associated transcription factors are expressed in human brain AVMs. A, Vertical scatter plots of KLF4, SNAI1, SNAI2, TWIST1, TWIST2, and ZEB1 expression in normal brain (NB), AVM‐1‐5, and CCM‐1‐4 by qRT‐PCR. Each point represents a unique sample and each value is represented as the mean ± SD (n = 3). The horizontal line indicates the median. B, Representative images of SNAI1/2 expression by IHC in NB, AVMs, and CCMs. Tissue within dashed boxes is shown at higher magnification to the right. Arrows indicate regions of PECAM‐/SNAI1/2‐positive cells, whereas arrowheads indicate SNAI1/2‐positive cells present in the perivascular tissue. Scale bar for a‐d, g, h, k, and l, 100 μM; scale bar for e, f, i, j, m, and n, 50 μM

EndMT is initiated by a series of TFs including KLF4, SNAI1, SNAI2, TWIST1, TWIST2, and ZEB1.^23^ To determine if these TFs were expressed, we profiled normal brain (NB), AVMs, and CCMs by qRT‐PCR. CCM tissue was analyzed in parallel, as EndMT is a known contributor to this disease. NB expressed KLF4, SNAI1, SNAI2, and TWIST1 but generally at lower levels than those observed for both AVM and CCM tissue (Figure 1A). NB expressed relatively high levels of ZEB1, consistent with expression reported in the Human Protein Atlas,^24^ but there was no detectible expression of TWIST2. CCMs showed heterogeneous expression of all six EndMT‐associated TFs, as has been reported previously.^18^ EndMT‐associated TFs were also observed in AVMs, at levels that were similar to or greater than those observed in CCM tissue. We also examined protein levels of SNAI1/2 in serial sections by IHC. As illustrated in Figure 1B, there was low expression of SNAI1/2 throughout NB, as predicted by qRT‐PCR. In AVM and CCM tissue, SNAI1/2 protein was observed throughout PECAM‐positive EC regions. We observed more SNAI1/2‐positive cells proximal to the dilated vessel lumens in AVMs as compared to CCMs.

**FIGURE 2:**
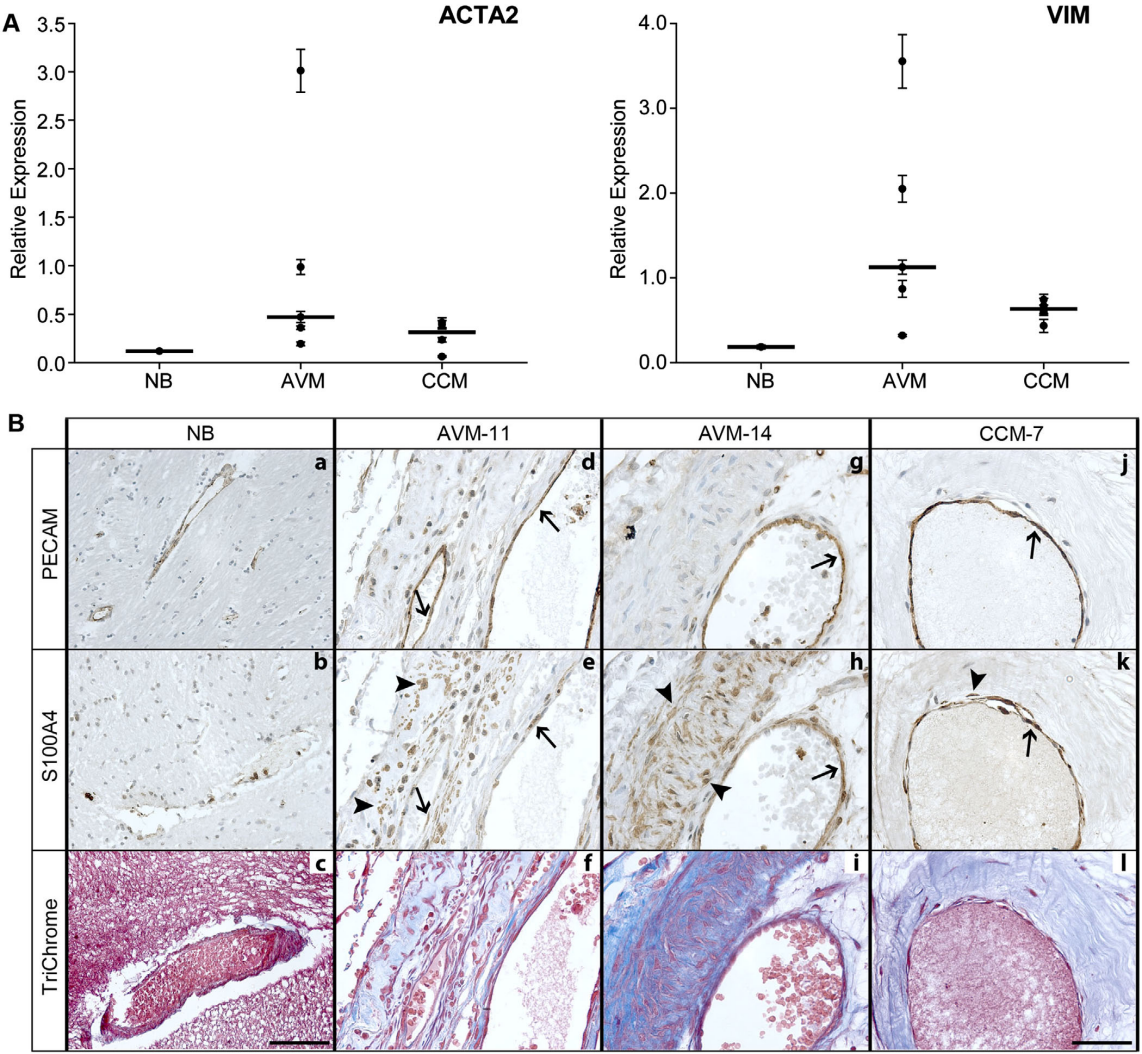
Expression of EndMT markers in human brain AVMs. A, Vertical scatter plots of expression of ACTA2 and VIM by qRT‐PCR in AVM‐1‐5 and CCM1‐4, compared to normal brain (NB). Each point represents a unique sample, with the sample value represented as the mean ± SD (n = 3). The horizontal line indicates the median. B, S100A4 is highly expressed in AVMs in PECAM‐positive ECs (arrows) as well as in the perivascular tissue (arrow heads) (d, e, g, and h). CCM tissue expressed S100A4, primarily in the PECAM‐positive ECs (arrows), although some S100A4 was observed proximal to the vessel lumen (arrowhead) (j and k). Both AVMs and CCMs demonstrated collagen deposition (blue) throughout the lesions (f, i, and l), including in regions of S100A4‐positive cells. NB expressed low levels of S100A4 and collagen (a‐c). Scale bar for a‐c, 100 μM; scale bar for d‐l, 50 μM

Progression of EndMT is associated with expression of mesenchymal proteins, including ACTA2, Vimentin, and S100A4, and deposition of extracellular matrix proteins. Consistent with previous work, we observed robust expression in AVMs of both ACTA2 and VIM, genes with roles in the cytoskeleton (Figure 2A).^25^, ^26^ We also examined expression of S100A4, a mesenchymal‐marker associated with EndMT, which regulates motility, cell cycle, and epithelial‐to‐mesenchymal cell transition (EMT; a process similar to EndMT).^27^ S100A4 was expressed in AVM tissue in both PECAM‐positive ECs regions and in regions proximal to the vessel (as shown in representative images in Figure 2B). S100A4 was primarily restricted to PECAM‐positive regions in CCMs. NB expressed low levels of S100A4. Throughout the perivascular regions in AVM tissue, we noted the presence of various cell morphologies in PECAM‐, SNAI1‐, and S100A4‐positive cells that included both classic rounded cells as well as spindle‐shaped, myofibroblast‐like cells.

To identify changes in ECM, we examined deposition of collagen, a structural protein associated with fibrogenesis and EndMT.^28^ As shown in representative images in Figure 2B, NB tissue contained low levels of collagen, mainly located around the vessels. In contrast, AVMs had robust collagen deposition, including in regions of co‐localization with S100A4, similar to that observed in CCMs. In general, collagen deposition correlated with S100A4 positive regions of the AVMs, and there were only rare instances of deposition without S100A4. There was limited S100A4 expression in regions that had no collagen.

### 3.2 PAI‐1 (Serpine1) is highly expressed in AVMs

**FIGURE 3:**
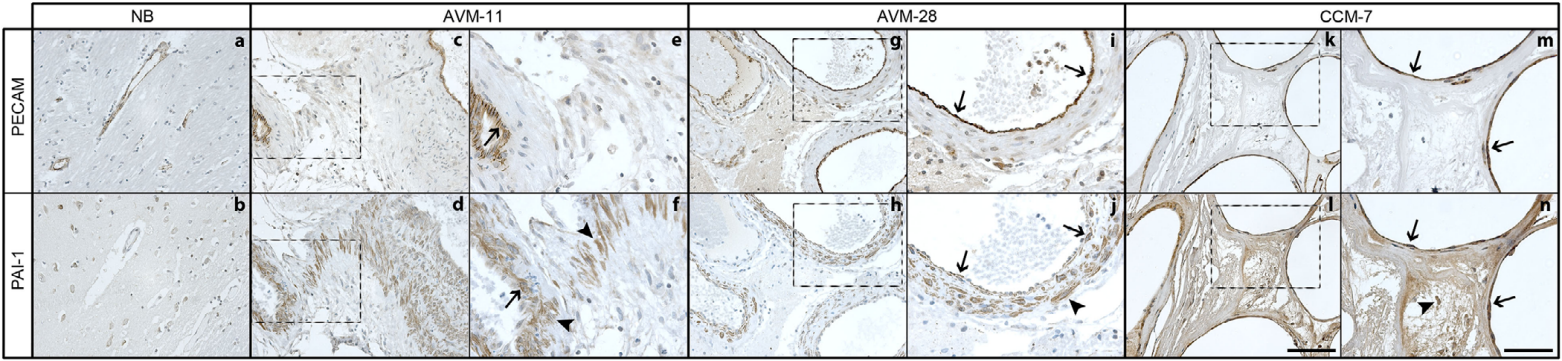
AVMs expressed PAI‐1, a protein transcriptionally regulated by TGF‐β. PAI‐1 was present in both PECAM‐positive ECs within the AVMs (arrows) and in PECAM‐negative regions (arrowheads) surrounding the vessels (c‐j). PAI‐1 was expressed throughout the CCM tissue (k‐n). Normal brain (NB) expressed low levels of PAI‐1 (a‐b). Regions in dotted boxes are shown at higher magnification to the right. Scale bar for a‐d, g, h, k, and l, 100 μM; scale bar for e, f, i, j, m, and n, 50 μM

Given the observed collagen deposition in AVMs and that EndMT is associated with increased fibrosis and ECM changes, we examined expression of PAI‐1, a protein that functions primarily to inhibit fibrinolysis, is transcriptionally regulated by TGF‐β, and has an established role in EndMT and EMT.^29^, ^30^, ^31^, ^32^ Although there was minimal PAI‐1 expression in normal brain (Figure 3b), AVMs and CCMs expressed abundant PAI‐1 in both PECAM‐positive regions and throughout the lesions, as shown for representative AVMs (Figure 3 [d, f, h, and j]) and CCM (Figure 3 [l and n]).

### 3.3 SMAD‐dependent TGF‐β signaling in AVMs

**FIGURE 4:**
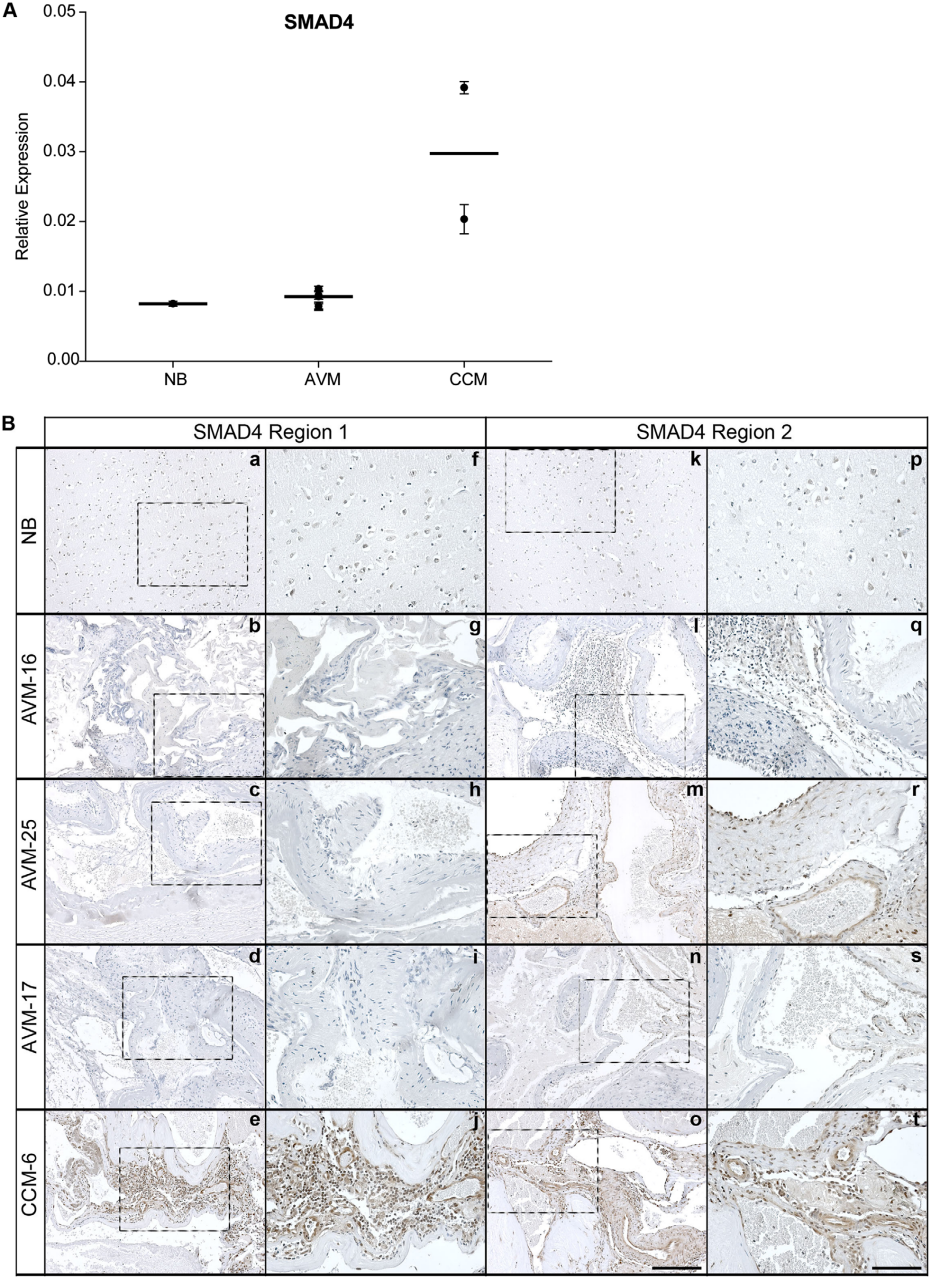
SMAD4 expression in AVMs. Normal brain (NB) and AVM1‐5 samples expressed similarly lower levels of SMAD4 mRNA by qRT‐PCR, as compared to CCM‐1‐2 samples, as shown in the vertical scatter plot (A). Each point represents a unique sample, with the sample value represented as the mean ± SD (n = 3). The line indicates the median. B, SMAD4 protein expression by IHC was weak in the AVM samples and limited to discrete regions of the tissue, whereas CCMs expressed robust SMAD4 protein. NB showed low expression throughout the sample. Region 1 and Region 2 indicate two areas of the tissue that is representative of the expression pattern for that sample. Tissue within dashed boxes is shown at higher magnification to the right. Scale bar for a‐e and k‐o, 100 μM; scale bar for f‐j and p‐t, 50 μM

As TGF‐β is a primary transcriptional regulator of PAI‐1 and a mediator of EndMT in CCMs, we determined if this signaling pathway also played a role in AVM pathology. We screened five AVMs for TGFBR1 (Alk5), ACVRL1 (Alk1), ACVR1b (Alk4), ACVR1C (Alk7), BMPR1A (Alk3), BMPR1B (Alk6), TGF‐β1, TGF‐β2, and TGF‐β3 by qRT‐PCR. As shown in Figure S1, AVMs expressed all of these genes, with the exception of TGF‐β3 that was below the limits of detection for all samples. To determine if there was activated TGF‐β signaling, we examined the presence of receptor‐regulated (R)‐SMADs, their corresponding activated phosphorylated forms (pSMADs), and co‐SMAD4 in eight AVMs by western blot analysis (SMAD1, SMAD5, pSMAD1/5, SMAD2, pSMAD2, SMAD3, pSMAD3, and SMAD4). Figure S2 illustrates the low expression of pSMAD1/5 and pSMAD3, and the weaker expression of pSMAD2. SMAD4 expression, however, was at or below the limits of detection, and only one sample (AVM‐37) had a reasonable signal. Given that co‐SMAD4 is required for pSMAD translocation into the nucleus, we further confirmed SMAD4 expression in NB, AVMs, and CCMs by qRT‐PCR and IHC. As shown in Figure 4A, NB and AVM tissue expressed similarly lower levels of SMAD4 mRNA compared to CCMs, consistent with the previously reported activated TGF‐β signaling in CCM tissue.^17^, ^18^ NB expressed low levels of SMAD4 protein by IHC, shown in two representative regions of the tissue (Region 1 and Region 2) (Figure 4B [a, f, k, and p]). SMAD4 protein expression in AVMs was heterogeneous with areas of both weak and high expression within the same AVM (Figure 4B [b, g, l, and q; c, h, m, and r; d, i, n, and s]). We observed high SMAD4 protein levels throughout the CCMs (Figure 4B [e, j, o, and t]). Taken together, this suggests that although there was some evidence for the expression of phosphorylated forms of (R)‐SMADs, SMAD‐dependent signaling may not be the primary signaling mechanism throughout the AVM lesion, given the low abundance of co‐SMAD4 mRNA and the localized, heterogeneous co‐SMAD4 protein expression.

### 3.4 Isolation of myofibroblast‐like cells from AVMs

Although PECAM‐ and αSMA‐positive cells have previously been isolated from human AVMs, we asked if there was an as‐yet‐unidentified proliferating cell population in AVMs. We isolated a unique population of proliferating cells from AVMs using a protocol optimized for isolating human neural progenitors.^22^ We generated cell lines cultured in serum‐free, minimally supportive NBA medium from three unique human AVMs located in typically nonproliferative brain regions. These cell lines proliferated beyond passage 20 (P20), whereas mature, differentiated cell types such as HUVECs and SMCs did not survive beyond 7 days under these same culture conditions, owing to the requirement for serum and other supplements. Figure S3 shows representative bright field images of the AVM cell lines, as well as HUVECs, SMCs, and attached neural progenitors for comparison.

**FIGURE 5:**
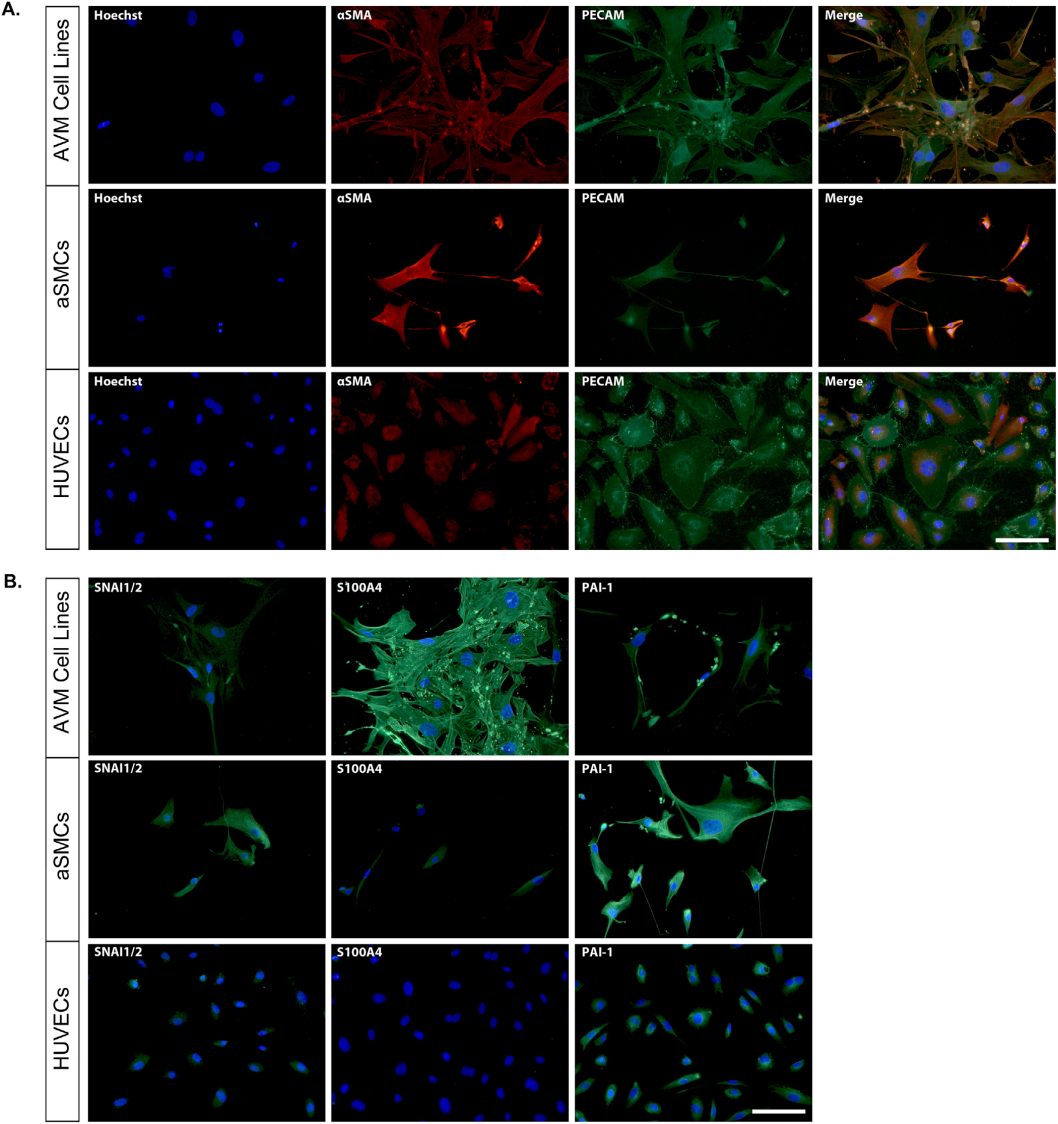
Protein profiling of AVM cell lines by immunocytochemistry. Expression of (A) mature cell‐type proteins, αSMA and PECAM, and (B) EndMT‐associated proteins SNAI1/2, S100A4, and PAI‐1, in AVM cell lines, aSMCs and HUVECs. Hoechst was used to label nuclei. Scale bars, 100 μm

To further characterize these cells, we examined the expression of proteins associated with mature vascular cell types and with EndMT by immunocytochemistry. PECAM, an EC‐specific protein, was expressed at low levels or not detected in AVM cells lines and SMCs, whereas the SMC‐specific protein, α‐SMA, was present in SMCs but was also observed in AVM cell lines. HUVECs expressed low levels of α‐SMA (Figure 5A). There were detectible levels of SNAI1/2 protein in both AVM cell lines and SMCs, but faintly observable in HUVECs (Figure 5B). S100A4 was strongly expressed in AVM cell lines, with only minimal immunoreactivity in SMCs and HUVECs. We observed high levels of PAI‐1 protein in SMCs, as well as in AVM cell lines, whereas HUVECs expressed low but detectible levels.

### 3.5 AVM cell line‐conditioned medium increases proliferation

**FIGURE 6:**
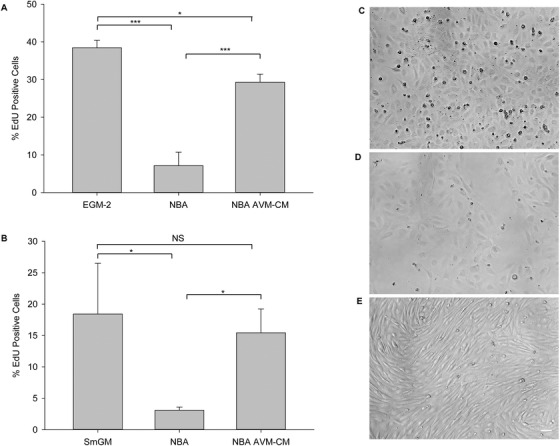
Serum‐free NBA AVM‐CM supported proliferation of HUVECs and SMCs, and induced phenotypic changes in HUVECs. HUVECs (A) and SMCs (B) were grown in EGM‐2 and SmGM, (respectively), serum‐free NBA, and serum‐free NBA AVM‐CM for 24 h. Random fields were scored for total number of cells (Hoechst 33342‐positive) and of proliferating cells with EdU incorporation. Representative bright field images of HUVECs grown in EGM‐2 (C), unconditioned, serum‐free NBA (D), and serum free NBA AVM‐CM (E). Error bars: mean ± SD; *T*‐test was performed to determine significance: ^*^ *P* ≤ .05; ^**^ *P* ≤ .001; scale bar, 200 μm

To determine if the AVM cell lines were capable of affecting mature cell types, we exposed HUVECs and SMCs to serum‐free AVM‐conditioned medium (NBA AVM‐CM). The conditioned medium was capable of increasing proliferation of both HUVECs and SMCs to rates approaching those observed in normal serum‐ and supplement‐containing growth medium (Figures 6A and 6B, respectively). NBA AVM‐CM was also capable of dramatically altering HUVEC phenotype, from the typical “cobblestone” pattern (Figure 6C) to a more spindle‐shaped, myofibroblast‐like morphology following 24 h in culture (Figure 6E). HUVECs did not thrive in unconditioned, serum free NBA (Figure 6D).

To gain an understanding of secreted cytokines that may be responsible for these effects, we analyzed NBA AVM‐CM, compared to NBA exposed to HUVECs (NBA HUVEC‐CM) and SMCs (NBA SMC‐CM), using a Luminex Human 62‐plex cytokine platform. AVM cell lines secreted a profile of cytokines that partially matched that of HUVECs and that of SMCs but also had a unique expression profile for VCAM1, LIF, HGF, BDNF, and VEGF (Figure S4).

## 4 DISCUSSION

Taken together, our research supports the role of EndMT in AVM disease. Although no single protein defines EndMT, we observed the presence of EndMT‐associated TFs and markers, collagen deposition, and expression of PAI‐1. Previous research provides additional support for this process, including increased expression of α‐SMA, laminin, and collagen and the presence of fibrosis in AVM tissue.^10^, ^26^, ^33^, ^34^ A microarray study of the nidus of a small number of AVMs reported increased expression of S100A4 and extracellular matrix proteins, including collagens, laminin, and fibronectin.^35^ Several groups have also described compromised vascular integrity (“vascular leakiness”), with the deposition of hemosiderin, reduced pericyte coverage, “silent” microhemorrhages, and undifferentiated, immature vasculature.^33^, ^36^, ^37^, ^38^, ^39^ A recent paper by Wang et al highlighted altered regulation of proteins involved in cell‐to‐cell communication, including gap junctions, tight junctions, and focal adhesions, further suggesting an abnormal vascular bed as would be expected with EndMT.^40^ We found S100A4 expression was not entirely correlated with collagen deposition in AVMs and may suggest the presence of other, as yet unidentified, cell types. S100A4 protein is also expressed by macrophages, fibroblasts, and activated lymphocytes, and has been implicated in cancer biology and in inflammatory diseases.^41^ We also observed some limited collagen deposition in S100A4‐negative regions, possibly related to a wound response due to clinically undiagnosed silent microhemorrhages or to previous treatment. Spindle‐shaped, Vimentin‐positive, myofibroblast‐like cells have also been described in AVM tissue both in, and outside of, the context of gamma knife treatment.^25^, ^37^, ^42^ Uranishi et al reported the presence of α‐SMA‐positive, spindle‐shaped cells in the perivascular space and decreased expression of Smoothelin, suggesting reduced contractibility in AVMs.^43^ Upregulation of EndMT‐associated genes was also reported in mutated KRAS (KRAS^G12V^)‐overexpressing HUVECs, a somatic mutation that is associated with AVMs.^5^ The precise identity of the proliferative, myofibroblast‐like cells we isolated from AVM tissue remains unclear, however. These cells expressed a range of proteins, subsets of which were shared by mature cell types, and secreted a cocktail of molecules capable of inducing proliferation and phenotypic changes in HUVECs. Although all three separate cultures were similar in protein expression, there was the potential for the introduction of in vitro culture artifacts and the subsequent loss of relevance to in vivo AVMs. Further definition of the AVM cell lines may provide new insight into their identity and highlight additional markers for sorting cells from AVM tissue.

PAI‐1 is a secreted glycoprotein that modulates extracellular proteolysis, tissue remodeling, and cell detachment, and is involved in EndMT.^32^, ^44^ We observed PAI‐1 protein in human AVMs, providing evidence of EndMT; however, this protein is expressed not only by myofibroblasts but also by neutrophils, astrocytes, and macrophages, further suggesting a complex cellular microenvironment within AVMs. Although TGF‐β is a well‐studied transcriptional regulator of PAI‐1, we found evidence for limited SMAD‐mediated TGF‐β signaling in AVMs. In support of this finding, Hauer et al also observed decreased SMAD4 expression in human brain AVMs by RNA‐seq,^45^ and in a rodent model of HHT, SMAD4 knockout resulted in brain AVMs.^46^ PAI‐1 expression is regulated by other processes, however, including canonical Wnt/β‐catenin signaling in human kidney tubular epithelial cells.^47^ PAI‐1 is significantly increased in a model of vascular malformations following postnatal brain EC‐specific deletion of Rbpj, a transcriptional mediator of Notch signaling, which the authors suggest may be as a result of crosstalk with TGF‐β signaling.^48^ Noncanonical TGF‐β signaling may contribute to AVMs, as might the MAPK‐ERK pathway, as has been shown in CCMs.^5^ Sonic Hedgehog signaling has also recently been implicated in human brain AVMs, supporting our previous findings of increased COUP TFII and expression of hedgehog family members in AVMs.^49^ Additional work is required to further understand the signaling landscape and how these diverse signaling cascades might converge to lead to AVMs.

The role of TGF‐β has been established in EndMT‐mediated fibrosis in many diseases, including cancer, cardiac and pulmonary fibrosis, and CCM pathology. Although our work suggests SMAD‐dependent TGF‐β signaling may not be the predominant mechanism, AVMs are recognized as heterogeneous lesions and our observation that SMAD4 is regionally present in AVMs suggests there may also be heterogeneity in signaling mechanisms. EndMT is also induced by other mechanisms, including disrupted flow, hypoxia, and inflammation. Increased inflammation may occur as a result of spatially nonuniform wall shear stress that is observed in the AVM vessels, ranging from 29 to 72 dynes/cm, significantly higher than normal vessels.^50^, ^51^ Evidence of inflammation in AVMs includes reports of increased expression of E‐selectin, VCAM‐1, ICAM‐1, and other pro‐inflammatory cytokines.^52^, ^53^ Interestingly, AVM cell line‐conditioned medium had increased levels of VCAM‐1 compared to both HUVEC‐ and SMC‐conditioned medium. Previously, we found AVMs expressed high levels of β macroglobulin (B2M), a component of the class 1 major histocompatibility complex further supporting a role for inflammation.^3^ The presence of macrophages, lymphocytes, and neutrophils also strongly suggests an active immune response in brain AVMs.^34^, ^54^, ^55^ Using an RNA‐seq approach in human brain AVMs, Hauer et al found increased expression of genes involved in inflammation and in the cytoskeleton and migration.^45^ Taken together, this work may present new pharmacological approaches for AVMs, given the compounds available to modulate fibrosis, EndMT, and inflammation. A clinical trial is currently underway using the immunosuppressant and antiproliferative drug, Sirolimus (Rapamune), in patients with severe AVMs (ClinicalTrials.gov Identifier: NCT02042326). An interesting question arises as to how EndMT contributes to CCM and AVM disease, but yet also contributes to cancer and fibrosis, diseases with vastly different consequences.

One of the limitations of this study was the inability to define the initiating and subsequent events in AVM development, as human AVM tissue was obtained at varying and uncontrollable points in disease progression. In addition, intact AVM tissue is technically difficult to manipulate on a molecular level, presenting challenges in dissecting the underlying signaling mechanisms. Future work, including the development of additional animal and in vitro human models, may shed light on these important questions.

## CONFLICT OF INTEREST

The authors declare no conflict of interest.

## Supporting information

Supporting Information.
