# Supplementary material for: Evidence for endothelial‐to‐mesenchymal transition in human brain arteriovenous malformations
Source: Clin Transl Med. 2020 Jun 21;10(2):e99. doi: 10.1002/ctm2.99 (PMC7403663; doi:10.1002/ctm2.99)
Supplement: Supplementary file 1 — Supporting Information. [file CTM2-10-e99-s001.docx]

**Online Supplemental Material:**


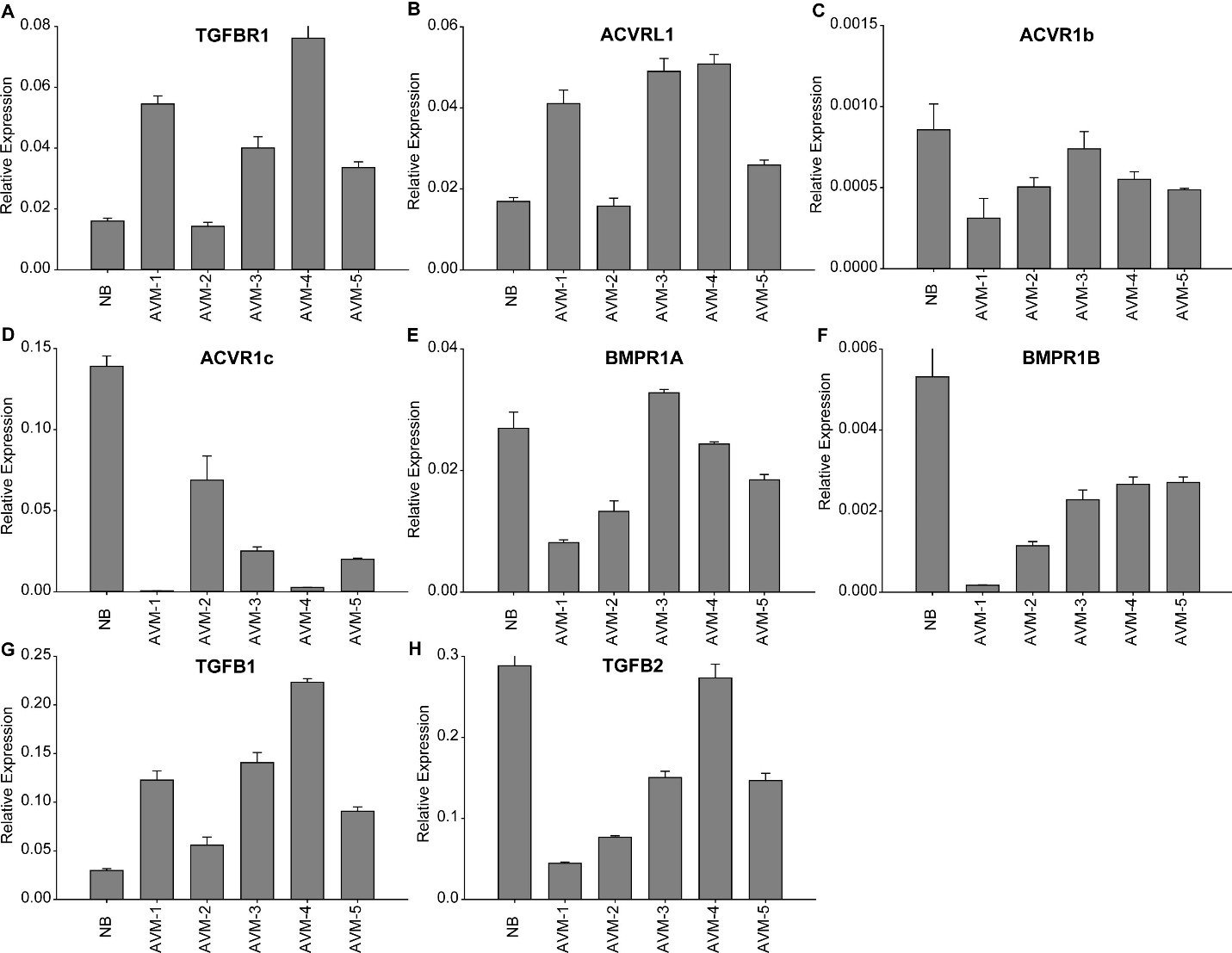


**Supplemental Figure 1.** Human brain AVMs express members of the TGFβ family of receptors and ligands by qRT-PCR. We examined normal brain (NB) and five AVM samples by qRT-PCR for the following gene expression: (A)TGFBR1 (Alk5); (B)ACVRL1 (Alk1); (C)ACVR1b (Alk4); (D)ACVR1c (Alk7); (E)BMPR1A (Alk3); (F)BMPR1B (Alk6); (G)TGF-β1; (H)TGF-β2. TGF-β3 was not detected in the normal or pathological samples. Values represent the mean ± SD (n=3).


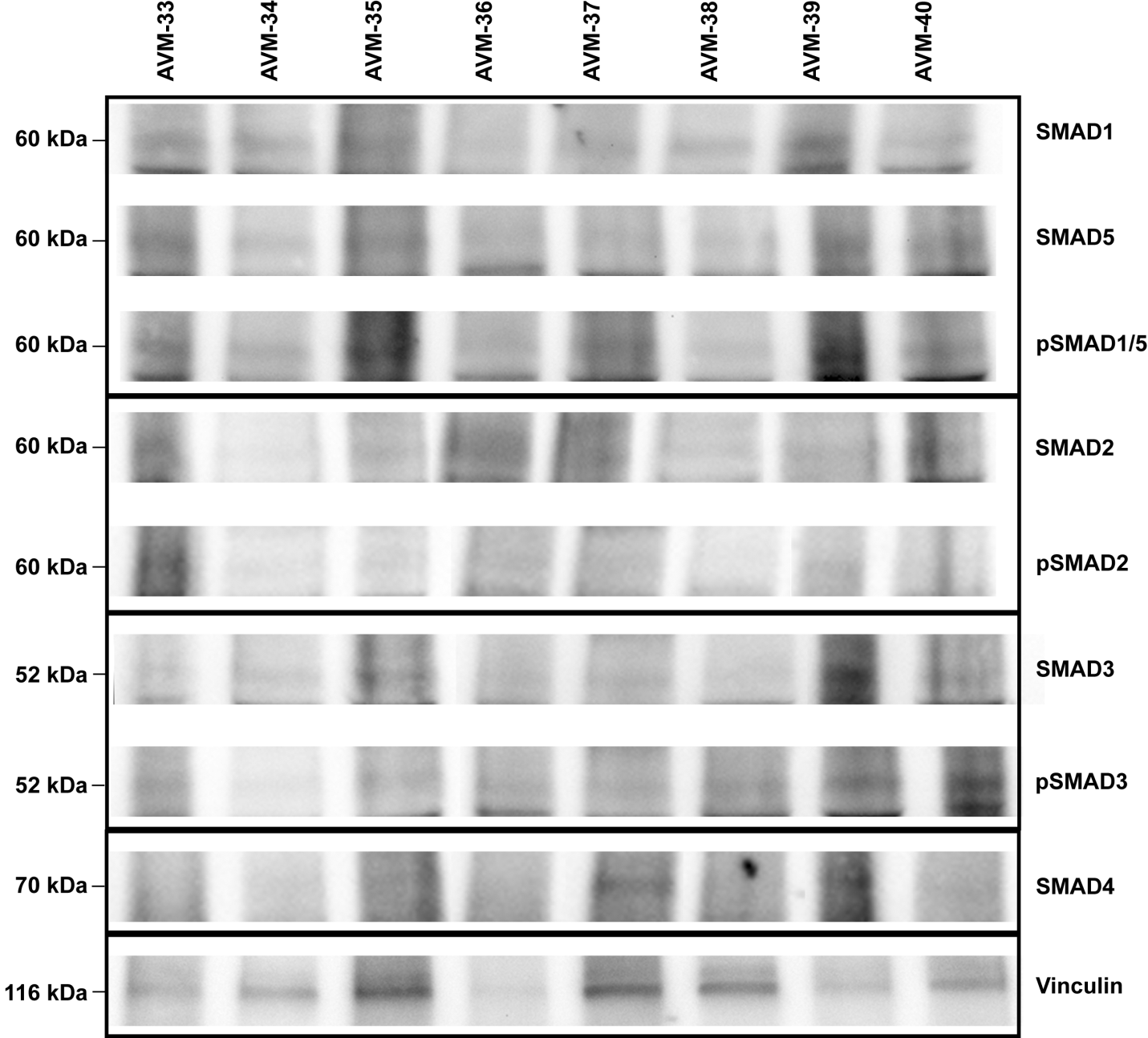


**Supplemental Figure 2.** Protein expression of SMADs and phosphoSMADs in whole human brain AVMs. Eight AVMs (AVM-33-40) were examined for the presence of SMADs and respective pSMAD using western blot analysis. The apparent molecular weights of each are labeled on the left of the panel. Vinculin used as a loading control. There was no post-imaging processing.


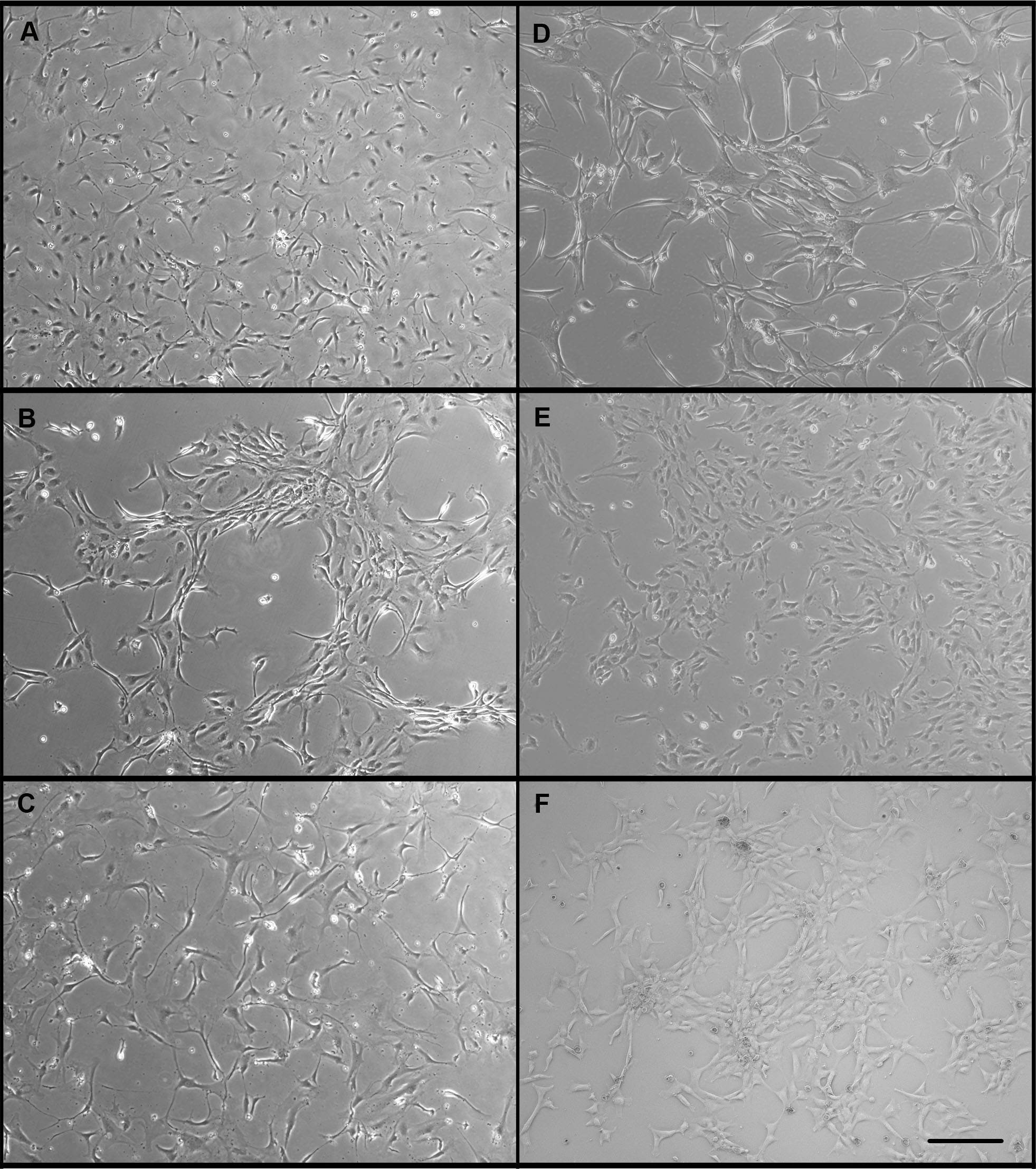


**Supplemental Figure 3.** Proliferating, adherent AVM cell lines were cultured in serum-free NBA medium. Representative bright field images of three separate human AVM cell lines all at P5 (A, B, C), human aSMCs P7 (D), HUVECs P7 (E), and attached human neural progenitors P28 (F). Scale bar, 500 µM.


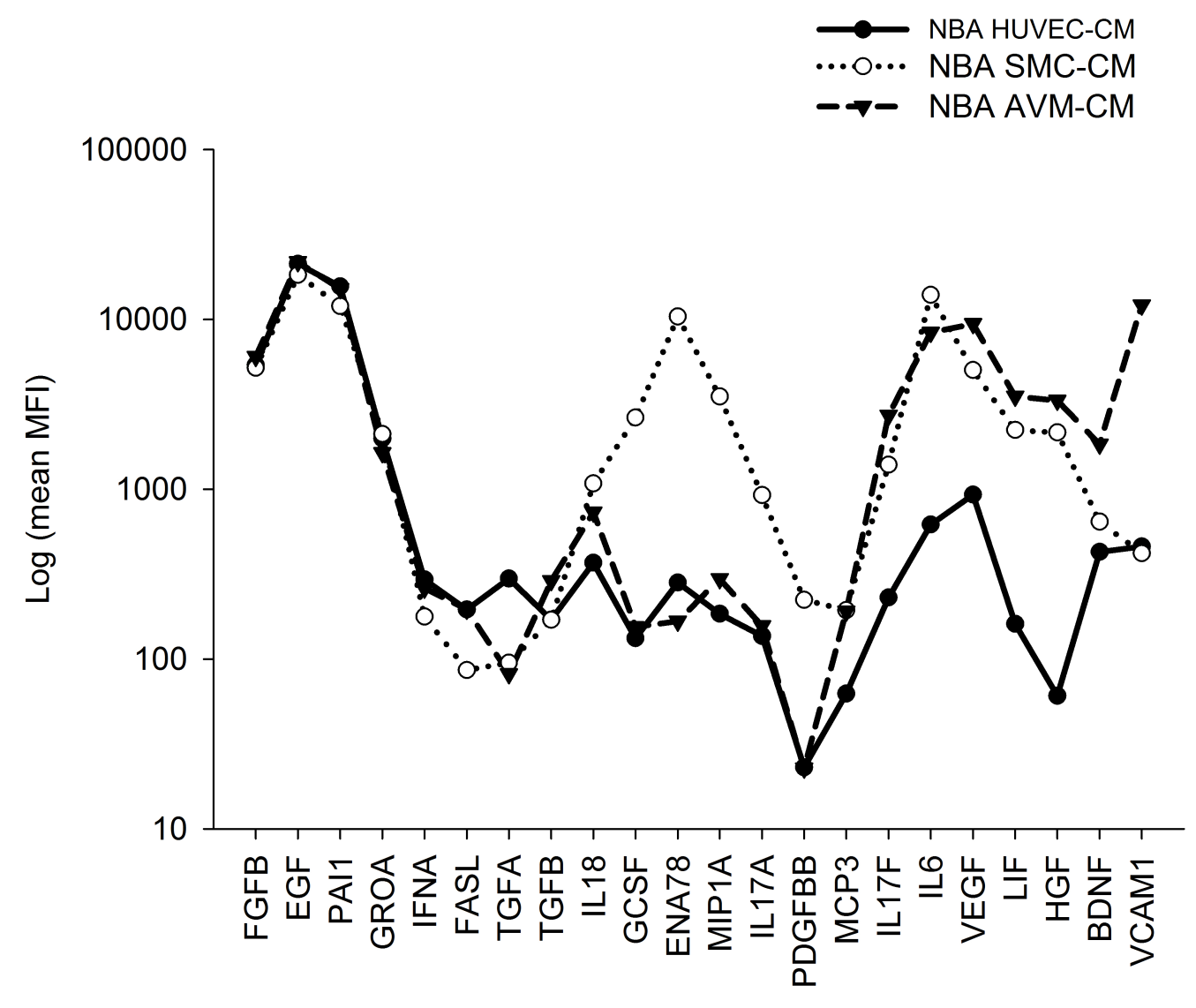


**Supplemental Figure 4.** Cytokine expression in conditioned medium (CM) of SMCs, HUVECs and AVM cell lines exposed to NBA medium. AVM cell lines expressed a cytokine profile that was similar to HUVECs in some aspects to that of HUVECs and Conditioned medium from SMCs, HUVECs and AVM cell lines grown in NBA-conditioned medium (CM) was analyzed to the , NBA SMC-CM and NBA AVM-CM. MFI (median fluorescence intensity) was measured from duplicate wells. AVM-CM expressed a unique cytokine profile that shared aspects of the HUVEC profile and aspects of the SMC profile.

|  | **qRT-PCR Expression** | **Western Blot Expression** | **IHC**  **Expression** | **AVM-derived Cell Lines** |
| --- | --- | --- | --- | --- |
| **AVMs:** |  |  |  |  |
| Number and Gender of Subjects (F/M) | 5 (2/3) | 8 (6/2) | 29 (13/16) | 3 (2/1) |
| Median Age at Surgery ± SD (y) | 43 ± 18 | 32 ± 13 | 26 ± 14 | 21 ± 10 |
| Acute Hemorrhage | 2 | 2 | 15 | 0 |
| SM Grade: 1 | 0 | 1 | 14 | 1 |
| 2 | 1 | 1 | 8 | 2 |
| 3 | 2 | 3 | 4 | 0 |
| 4 | 1 | 3 | 2 | 0 |
| 5 | 1 | 0 | 1 | 0 |
| Pre-surgery Treatment | E: 4  S: 1 | E: 4  S: 1  E, R: 1 | E: 7  S: 1  R, S: 1 | E: 1  R, S: 1  E, R: 1 |
| **CCMs:** |  |  |  |  |
| Number and Gender of Subjects (F/M) | 4 (3/1) | N/A | 4(2/2) | N/A |
| Median Age at Surgery ± SD (y) | 44 ± 14 | N/A | 43 ± 4 | N/A |

**Supplementary Table 1.** Summary of patient demographics and clinical history. Pre-surgery treatment included embolization (E), previous surgery (S) and radiosurgery (R). Tissue for qRT-PCR and Western blot analyses were obtained fresh frozen; for IHC, paraffin-embedded; for AVM-derived cell lines, fresh. SM = Spetzler-Martin Grade.

| **Gene** | **Catalog Number** | **Exome Spanning** |
| --- | --- | --- |
| GAPDH | Hs03929097_g1 | No |
| ACVRL1 | Hs00953798_m1 | Yes |
| ACVR1B | Hs00923299_m1 | Yes |
| ACVR1C | Hs00899854_m1 | Yes |
| BMPR1A | Hs01034913_g1 | Yes |
| BMPR1B | Hs01010965_m1 | Yes |
| TGFBR1 | Hs00610320_m1 | Yes |
| TFGB1 | Hs00998133_m1 | Yes |
| TGFB2 | Hs00234244_m1 | Yes |
| TGFB3 | Hs01086000_m1 | Yes |
| ACTA2 | Hs00426835_g1 | Yes |
| VIM | Hs00958111_m1 | Yes |
| KLF4 | Hs00358836_m1 | Yes |
| SNAI1 | Hs00195591_m1 | Yes |
| SNAI2 | Hs00161904_m1 | Yes |
| TWIST1 | Hs01675818_s1 | No |
| TWIST2 | Hs02379973_s1 | No |
| ZEB1 | Hs00232783_m1 | Yes |
| SMAD4 | Hs00929647_m1 | Yes |

**Supplementary Table 2.** List of FAM^TM^ qRT-PCR primer sets against human genes. All primers were purchased from ThermoFisher Scientific.
